# Seasonal Variation of Aflatoxin Levels in Selected Spices Available in Retail Markets: Estimation of Exposure and Risk Assessment

**DOI:** 10.3390/toxins14090597

**Published:** 2022-08-29

**Authors:** Farah Naz, Francis Verpoort, Shahzad Zafar Iqbal, Nadia Naheed, Muhammad Rafique Asi

**Affiliations:** 1 Institute for Advanced Study, Shenzhen University, Shenzhen 518060, China; 2 College of Physics and Optoelectronic Engineering, Shenzhen University, Shenzhen 518060, China; 3 Laboratory of Organometallic, Catalysis and Ordered Materials, State Key Laboratory of Advanced Technology for Materials Synthesis and Processing, Wuhan University of Technology, Wuhan 430070, China; 4 Ghent University Global Campus, Incheon 406-840, Korea; 5 National Research Tomsk Polytechnic University, 634050 Tomsk, Russia; 6 Department of Applied Chemistry, Government College University, Faisalabad 38000, Pakistan; 7 Food Toxicology Lab, NIAB, Jhang Road, Faisalabad 38000, Pakistan

**Keywords:** AFB1, AFs, selected spices, dietary intake, tocopherol levels, seasonal variations

## Abstract

A total of 603 samples of selected spices from different seasons (winter and summer) were analyzed for the occurrence of aflatoxin B_1_ (AFB_1_), total AFs, and tocopherols. The findings revealed that 120 (38.7%) samples from the summer and 136 (46.4%) samples from the winter season were observed to be infected with AFB_1_ and a large amount of AFs. The highest means of both AFB_1_ and total Afs were observed in red pepper, i.e., 15.5 ± 3.90 µg/kg and 22.90 ± 4.10 µg/kg, respectively. The minimum averages of AFB_1_ and total AFs were observed in cloves of 6.32 ± 1.8 and 8.40 ± 1.60 µg/kg, respectively (from the winter season). The seasonal variations in the levels of the total AFs in selected spices were observed to be nonsignificant (*p* ≥ 0.05), except for the levels in red pepper and ginger samples, which showed significant differences (*p* ≤ 0.05). The maximum average of the dietary intake of Afs, 4.80 µg/day/kg, was found in ginger from the winter season in individual females. Furthermore, the findings document that the maximum level of total tocopherol, i.e., 44.8 ± 9.3 mg/100 g, was observed in black pepper from the winter season. A significant difference in the concentration of total tocopherols was observed in selected spices from the summer and the winter seasons (*p* ≤ 0.05).

## 1. Introduction

Spices are essential food ingredients and defined as “the eatable parts of plants which are usually mixed with food items to create color, aroma, appearance, and also for flavoring purposes” [1]. Spices such as paprika, oregano, clove, onion, and garlic have characteristic antimicrobial activities [2]. The essential spices such as chili, coriander, turmeric, cinnamon, cloves, ginger, black pepper, and nutmeg are famous for their food products. In a report from 2016–17, 2.8 million tons of spices were produced worldwide, and 95% of production was contributed by Asia, 0.5% by Europe, 2.9% by Africa, and a 1.5% share from America [3]. In a report by the Food and Agriculture Organization, spices were harvested from an area of 16,653 ha in 2019, as compared to 17,153 ha in 2018 [4], with a production of 76.08 tons as compared to the production of 77.54 tons in 2018. Chilies and dry peppers were harvested in 2018 and 2019 from areas of 47,349 and 65,275 ha, with a production of 101.66 thousand tons and 148.14 thousand tons, respectively. Spices are exposed to various microflora during harvesting, processing, transportation, and storage, e.g., normally open grounds are used for sun-drying due to this being a cost-efficient and inexpensive approach. However, it creates substantial losses in terms of product quality and quantity [5]. Fungal infection in spices leads to mycotoxin formation, with temperature ranges from 25 to 30 °C and moisture contents greater than 16% [6]. Furthermore, the storage of crops under inadequately maintained on-farm or in-house requirements could also lead to damages to the value of crops, which results from insect infestation and fungal contamination [7]. Mycotoxins are recognized as toxic compounds that are formed by different types of foodborne fungi. They have shown poisonous and carcinogenic effects in animals and humans [7]. The fungi can attack fruits, cereals, grains, animal forages, and spices. The species Fusarium, Alternaria, Aspergillus, Penicillium, and Claviceps genera are important fungal species that produce mycotoxins [8]. The significant types of mycotoxins are aflatoxins (AFs), fumonisins, ochratoxin A, sterigmatocystin, zearalenone, patulin, and trichothecenes (HT-2 and T-2 toxins, nivalenol, and deoxynivalenol) [9]. The Food and Agriculture Organization documented that 25% of food and food crops are infected by these mycotoxins [10]. The main producers of AF fungi are *Aspergillus flavus* and *Aspergillus parasiticus*, and rarely by *A. nomious* [11,12]. AFs are odorless, tasteless, and invisible chemical compounds with polar solubility [13]. International organizations, such as The International Agency for Research on Cancer [14], have categorized these fungal metabolites as group 1 carcinogens to humans. The main subclass of AFs is aflatoxin B_1_ (AFB_1)_, which is identified as the most toxic of all [15]. The other classes include aflatoxin B2 (AFB2), aflatoxin G_1_ (AFG_1_), and aflatoxin G_2_ (AFG_2_). The exposure of aflatoxins to humans occurs in two ways: directly by consuming contaminated food or indirectly using produce from an animal or plant origin (e.g., eggs, meat, or vegetables) [16]. AFB_1_ consumed by animals through feed could cause lower blood production and anorexia [17]. It has been observed that chronic exposure to AFs could cause liver cancer. If the carrier has the hepatitis B virus, it synergistically deteriorates the health and functions of the liver. Acute aflatoxicosis causes episodic poisoning outbreaks and death [18,19,20]. The exposure of AFB_1_ in humans is found to increase the cellular calcium in mitochondria, which is responsible for increasing the levels of reactive oxygen species in cells [21,22]. However, aflatoxin M_1_ (AFM_1_) is the metabolite of AFB_1_, and it is mainly excreted in milk and the urine of milking animals [23]. A limit of 5 µg/kg for AFB_1_, and 10 µg/kg for total AFs in spices was implemented by the European Union (EU) [24].

Pakistan is a tropical country with severe environmental conditions. Therefore, the routine analysis for the presence of such toxins is highly desirable and recommended. Previous findings have documented a considerable concentration of ochratoxin A (OTA) and AFs in chilies [25,26,27,28,29,30,31,32,33]. Therefore, the current research is focused on investigating the seasonal variation and prevalence of AFB_1_ and total AFs in selected spices, comparing the amounts of AFB_1_ and total AFs with the European Union (EU) regulations, and investigating the daily intake of aflatoxins in spices and determining the levels of tocopherols in spices. The results will be helpful for law enforcement agencies and food regulation agencies to execute rigorous guidelines for these mycotoxins in spices.

## 2. Results and Discussion

### 2.1. Validation of Analytical Method

The HPLC method validation parameters were performed by evaluating the limit of detection (LOD) and the quantification limit (LOQ). The LOD and LOQ for AFB_1_ and AFG_1_ were 0.05 and 0.15 μg/kg, and 0.07 and 0.22 μg/kg for AFB_2_ and AFG_2_, respectively. The LOQ was assessed as having a signal-to-noise ratio S/N = 10 and an LOD as (S/N) = 3. The accuracy and precision of the method was determined using recovery analysis. The fortified amounts of 2, 10, and 25 μg/kg of AFB_1_ and AFG_1_ were mixed into spices, and the levels of 2, 8 and 20 μg/kg of AFB_2_ and AFG_2_ were combined in unaffected spice samples. The recovery values of all fortified samples ranged from 78.8% to 108.6%, with the RSD varying from 10 to 22%, as shown in Table 1. Reproducibility refers to producing the same results using different labs, and repeatability refers to producing the same results using the same analytical conditions.

### 2.2. Occurrence of AFs in Spices

**Figure 1:**
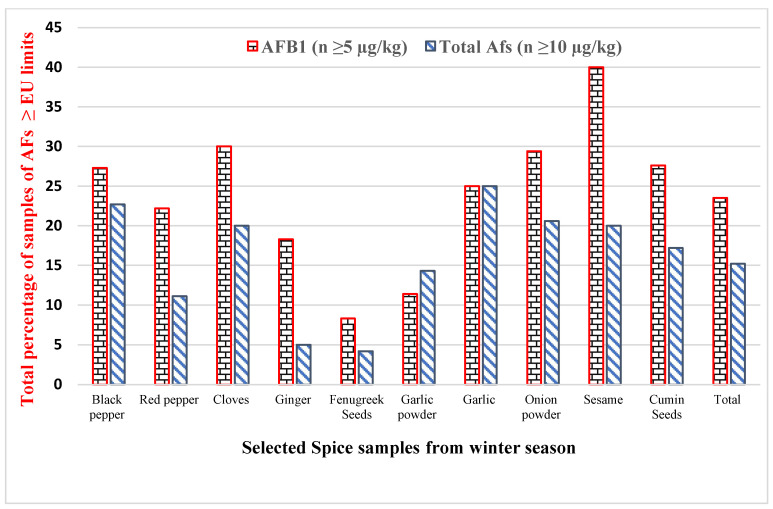
The percentage of samples higher than the recommended limits for AFB_1_ and total AFs in winter season.

**Figure 2:**
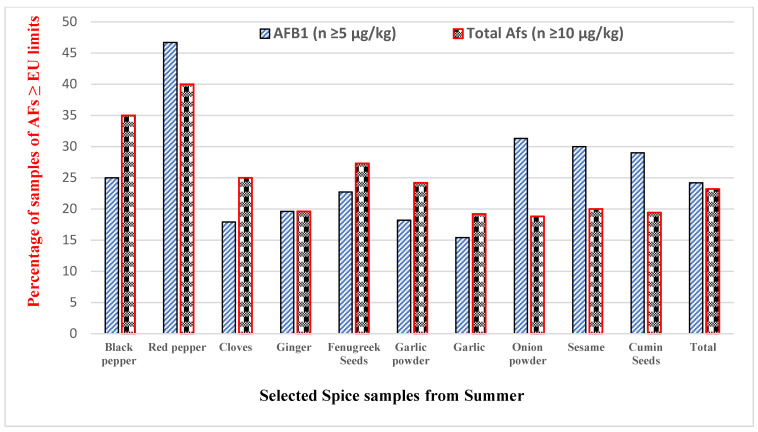
The percentage of samples higher than the recommended limits for AFB_1_ and total AFs in summer season.

The incidence and co-occurrence of AFs in different spices were examined in 293 samples (winter season) and 310 samples (summer season), as shown in Table 2. The research findings revealed that the highest averages of AFB_1_ and total AFs were observed in red pepper, i.e., 15.50 ± 3.90 µg/kg and 22.90 ± 4.10 µg/kg, respectively, and the minimum average levels were observed in cloves, 6.32 ± 1.80 and 8.40 ± 1.60 µg/kg, respectively, from the winter season. During the summer season, the highest average amounts of AFB_1_ and total AFs were discovered in red pepper, i.e., 9.80 ± 2.10 and 12.50 ± 1.90 µg/kg, respectively, and the minimum average amounts were documented in garlic, 4.10 ± 1.60 and 8.50 ± 1.60 µg/kg, respectively. The outcomes of the current survey show that the levels of total AFs in selected spices from different seasons were nonsignificant (*p* ≥ 0.05), except for the red pepper and ginger samples, which showed a significant difference (*p* ≤ 0.05). Furthermore, 136 (46.4%) samples from the winter season and 120 (38.7%) samples from the summer season were examined and shown to be positive with AFs. The percentages of samples above the mentioned limit of the EU for AFB_1_ (≥5 µg/kg) and total AFs (≥10 µg/kg) in selected spices were 24.2% and 23.2%, respectively, from the winter season, as shown in Figure 1. Similarly, the percentages of samples greater than the set limits (the EU) for AFB_1_ and total AFs from the summer season were 23.5% and 15.2%, respectively, as presented in Figure 2.

Earlier studies [34,35,36,37,38,39,40,41,42,43,44] from different countries (Table 3) showed the levels of AFs in spices. Our earlier study, Iqbal et al. [27], from Pakistan, reported that AFB_1_ levels were found in 39.7% samples of ground chili samples and 33.3% in the whole chili samples, i.e., with mean levels of AFB_1_ of 18.5 and 17.4 µg/kg, respectively. These results are in agreement with the findings of the current research.

The variations and fluctuations in the levels of AFs in spices are dependent on various factors. For example, temperature and moisture are the two main factors affecting the growth of mold, while factors such as dry, hot surface areas and the microbial resistance of crops against the toxigenic species are vital [6]. The toxicity level depends upon the difference in the toxin structure during farming seasons [45]. The high occurrence of AFs in spices might be due to the climatic conditions in Pakistan, which are conducive for the growth of aflatoxigenic fungi [39]. Furthermore, the old traditional technology and drying techniques used with spices are also one other reason for the high incidence of AFs in spices [46]. Pakistan is the major contributor in chili production and has tropical climate conditions, i.e., a hot and humid environment, for the growth of spices. Therefore, the production and postharvest conditions such as the processing, transportation, and storage of spices can lead to significant challenges in the quality of the food. To avoid or minimize the levels of AFs in food stuffs, adopting good harvesting practices and good storage practices, and implementing critical control point (HACCP) principles will be decisive for controlling the proliferation of fungi [30,32]. The following points should be kept in mind to avoid fungal attacks on spices.

- The proper handling of spices during storage and transportation with skilled practices.
- Drying process should be carried out under controlled conditions of temperature and moisture.
- Humidity levels should be controlled during preharvest and postharvest steps.

### 2.3. Estimation of Dietary Intake

Assessments of the dietary intake of the total AFs in spices were determined from different seasons and appear in Table 4. The highest mean dietary intake of AFs was observed in onion powder, 3.71 µg/day/kg, in individual females. However, the maximum average dietary daily intake of total AFs was found to be 2.91 µg/day/kg in onion powder in individual males. The highest average dietary intake of AFs was documented in ginger, i.e., 4.80 µg/day/kg, and the lowest in cloves from the winter season, i.e., 0.76 µg/day/kg, in the female participants. The maximum dietary intake levels were noticed in ginger, i.e., 3.77 µg/day/kg, and the lowest, 0.60 µg/day/kg, in cloves, in individual males.

The levels of dietary assessment from Pakistan were documented in another study, Akhtar et al. [47]. They documented the dietary intake of AFs in branded and nonbranded spices, and the observed dietary intake of AFs was 0.72 ng/kg bw/day, ranging from 0.31 to 1.30 ng/kg bw/day, in branded spices, and 1.59 ng/kg bw/day, ranging from 0.66 to 3.29 ng/kg bw/day, in nonbranded spices. Iqbal et al. [30] documented the maximum dietary intakes of AFB_1_, OTA, and total Afs, i.e., 3.67, 3.629, and 3.82 µg/day/kg in chili sauces, respectively, from Pakistan, which are comparable to the results of the dietary intake levels documented in the present study.

## 3. Conclusions

The study documents the high levels of AFB_1_ and total AFs in selected spices. The results show a nonsignificant difference between the total amount of AFs from the summer season to the winter season, except for the red pepper and ginger samples, which show a significant difference (*p* ≤ 0.05). The estimated dietary intake of total AFs is higher in individual females as compared to individual males. The results show that there is a significant difference in the tocopherol levels in selected spices from the winter season compared to the summer season. The results are useful for farmers, consumers, local traders, and law enforcement agencies to execute rigorous rules in the country.

## 4. Materials and Methods

### 4.1. Sampling

A total of 603 samples of selected spices (black pepper, red pepper, cloves, ginger, fenugreek seeds, garlic, garlic powder, onion powder, sesame, and cumin seeds) were collected from central cities of Punjab, Pakistan. Due to uneven distribution of fungi, a size of at least half kg was selected, and the samples were stored in polyethylene zip bags at room temperature in lab.

### 4.2. The Regents and Chemicals

The standards, including of Afs, tween^®^ 20, phosphate-buffered saline (PBS), acetonitrile (HPLC grade), and methanol (HPLC grade), were obtained from Sigma-Aldrich (Steinheim, Germany). The cleanup columns, immunoaffinity columns (IAC), AflaOchra (IAC) were acquired from VICAM (Watertown, MA, USA). Furthermore, other chemicals and reagents used during research were freshly prepared and of high purity (≥95%). Water (double-distilled) was preferred throughout the study.

### 4.3. Extraction Process for AFs

The samples were kept in a vacuum oven for 24 h till dryness, and then ground to a particle size (<0.5 mm) with the help of a grinding mill (Retsch, Haan, Düsseldorf, Germany). The process of extraction of AFs in spices was carried out following the method (AOAC official method 2008.02). The sample of 5 g was weighed, and 1 g of NaCl was added to a 25 mL solution of methanol in a beaker after adding 0.5% of NaHCO_3_ (70:30 *v/v*). Then the mixture was centrifuged (4500 rpm) for 10 min. After centrifugation, supernatant (7 mL) was diluted in 28 mL of 0.1% phosphate buffer saline (PBS) containing Tween^®^ 20 (1%). The solution (25 mL) was passed at a speed of 60 drops per minute through an IAC. Then washing was carried out on a column with a solution of 5 mL of PBS (10 mM), at a flow rate of one drop per second. Finally, the AFs were eluted by passing 2 mL of methanol with a speed, as mentioned above. The eluted solution was then moved in a glass vial and diluted with 1 mL of mobile phase before final analysis.

### 4.4. Analytical Method

The HPLC instrument used for analysis was a Shimadzu (LC-10A, Shimadzu, Kyoto, Japan). The HPLC column was nonpolar of C_18_ (Discovery HS) and the detector was a fluorescence detector (RF-530) used for AFs and tocopherols analysis. For tocopherols, gradient mobile phase with two solvents were used, i.e., solvent A (50% acetonitrile) and solvent B (50% methanol), at 1 mL/min flow rate. The detector (fluorescence) was used for tocopherol analysis with emission and excitation wavelengths of 325 nm and 295 nm, respectively. However, isocratic mobile phase with a combination of acetonitrile–methanol–water (20:20:60, *v/v/v*) was utilized for the evaluation of AF levels in selected spices with a flow rate of 1 mL/min. The wavelengths of excitation and emission were set at 360 and 440 nm, respectively. The final sample used for HPLC injection was 20 µL.

### 4.5. Estimation of Exposure

The estimated daily intake (EDI) was estimated as mentioned by FAO/WHO [48], with given formula

(1) $$\mathrm{Dietary}\;\mathrm{Intake}\;\left(\mathrm{EDI}\right)\;µg/\mathrm{kg}/\mathrm{day}=\frac{\mathrm{Consumption}\;\mathrm{of}\;\mathrm{Spices}\;\left(\mathrm{kg}\right)x\;\mathrm{AFs}\;\mathrm{average}\;\mathrm{level}\;µg/\mathrm{kg}\;}{Mean\;weight\;\left(kg\right)}$$

The consumption data were obtained from 500 individuals (males and females) including a food frequency questionnaire and asking them the portion of each spice used in their daily food dishes. The questionnaire was evaluated considering the uses of spice consumption in different food dishes during each day, in a week, and food dishes cooked occasionally. This questionnaire analyzed the exact amount of spices used in food products. The average weight of male and female participants was 70.

### 4.6. Margin of Exposure Characterization

The carcinogenic and toxic effects of AFs were determined based on margin of exposure Assessment (MOE), which was calculated by dividing the benchmark lower dose limit (BMDL) for aflatoxins as 400 ng/kg bw/day as expressed in Equation (2).

(2) $$\mathrm{MOE}=\frac{BMDL}{EDI}$$

Health risk of exposure to pollutants was determined using MOE (margin of exposure) [49,50]. MOE can be estimated using benchmark dose (BMD) or benchmark dose lower confidence limit 10% (BMDL10). BMD is defined as a dose that causes a low but measurable effect and BMDL10 is the dose that does not cause more than 10% cancer with 95% confidence. This was determined by dividing the benchmark dose lower limit (BMDL) for aflatoxins 400 ng/kg^−1^ bw/day^−1^ (reference value for AFB1 in control group) [51]. When MOE value is equal or greater than 10,000, it poses fewer health concerns, and if its value is less than 10,000, it indicates that exposure to that pollutant has high health risks [52].

### 4.7. Estimation of Liver Cancer Risk

The ingestion of AFs and the synergistic effects on liver cancer are well established. The liver cancer risk in Pakistani population were calculated by JECFA [53]. According to JECFA, the potency value for AFB_1_ corresponds to 0.3 cancers/year/100,000 population ng/kg bw/day, and the hepatitis positive rate is 2.4% [54]. The mean potency can be calculated as

Mean potency = [0.03 × HBsAg – negative individuals in Pakistan] + [0.01 × HBsAg − positive individuals/prevalence rate in Pakistan] = (0.03 × 0.024) + (0.01 × 0.976) = 0.00072 + 0.00976 = 0.0105(3)

The formula for cancer risk (cancer per year per 10^5^ individuals per ng/kg bw/day was estimated using formula

Cancer risk = EDI × Mean potency(4)

A carcinogenic risk of <10^−4^ is deemed acceptable (tolerable) by the US Environmental Protection Agency. However, if the risk is greater than 10^−4^, it is declared carcinogenic [54].

### 4.8. Statistical Analysis

The findings were given as mean levels using standard deviations. The standard curve (seven point) was created, and the equation of straight line was achieved. Using linear regression the coefficient of regression/correlation was obtained. To evaluate the significant difference among AFs in selected spices from seasonal variation, one-way ANOVA (α = 0.05) was used (SPSS, IBM SPSS Statistics 25, USA), and LSD was applied to obtain the significant difference among groups.

## Figures and Tables

**Table 1:** Analytical parameters for the analysis of AFs and tocopherols.

Target Compound	Linearity µg/kg	LOD µg/kg	LOQ µg/kg	R^2^	Precision (%RSD)	
					Reproducibility *	Repeatability *
AFB_1_	0.5–120	0.05	0.15	0.9940	10	15
AFB_2_	0.5–50	0.07	0.22	0.9920	14	17
AFG_1_	0.5–120	0.05	0.15	0.9870	13	9
AFG_2_	0.5–50	0.07	0.22	0.9890	15	17

RSD: relative standard deviation; LOQ: limit of quantification; LOD: limit of detection. * Reproducibility = (mean of 7 replicates); * Repeatability = (mean of 7 replicates).

**Table 2:** Occurrence of AFB_1_ and total AFs (µg/kg) in selected spices from summer and winter season from Punjab, Pakistan.

	Summer Season				Winter Season			
Spices	Samples	Positive Sample	AFB_1_	Total AFs	Samples	Positive Sample	AFB_1_	Total AFs
	n	n (%)	µg/kg ± SD	µg/kg ± SD	n	n (%)	µg/kg ± SD	µg/kg ± SD
Black pepper	22	10 (45.4)	5.60 ± 1.80	8.80 ± 2.10 ^NS^	20	12 (60.0)	7.90 ± 2.10	11.20 ± 2.50 ^NS^
Red pepper	18	8 (44.4)	9.80 ± 2.10	12.50 ± 1.90 **	15	10 (53.3)	15.50 ± 3.90	22.90 ± 4.10 **
Cloves	30	12 (40.0)	4.32 ± 1.00	6.50 ± 1.50 ^NS^	28	9 (32.1)	6.32 ± 1.80	8.40 ± 1.60 ^NS^
Ginger	60	15 (25.0)	9.42 ± 2.50	11.45 ± 2.10 **	56	19 (33.9)	14.50 ± 2.00	20.60 ± 4.40 **
Fenugreek Seeds	24	6 (25.0)	5.80 ± 1.90	8.60 ± 1.80 ^NS^	22	8 (36.3)	7.40 ± 1.95	10.20 ± 1.80 ^NS^
Garlic powder	35	10 (28.6)	6.72 ± 1.50	9.20 ± 1.90 ^NS^	33	12 (36.4)	8.90 ± 3.20	11.10 ± 3.40 ^NS^
Garlic	28	12 (42.8)	4.10 ± 1.60	8.50 ± 1.60 ^NS^	26	13 (50.0)	5.90 ± 2.40	11.20 ± 2.10 ^NS^
Onion powder	34	15 (44.1)	6.10 ± 1.20	10.20 ± 2.10 ^NS^	32	18 (56.2)	7.30 ± 1.90	11.90 ± 2.30 ^NS^
Sesame	30	18 (60.0)	7.90 ± 1.30	9.10 ± 2.20 ^NS^	30	19 (63.3)	9.50 ± 1.90	12.40 ± 2.0 ^NS^
Cumin Seeds	29	14 (48.3)	4.50 ± 1.40	10.50 ± 1.40 ^NS^	31	18 (58.1)	6.40 ± 1.80	11.90 ± 2.10 ^NS^
Total Samples	310	120 (38.7)			293	136 (46.4)		

N = number of samples. NS = nonsignificant (the samples show nonsignificant difference between summer and winter seasons (α = 0.05). ** = the samples show significant difference in AF levels in summer and winter seasons.

**Table 3:** Incidence and occurrence of AFs in different spices from different countries.

Food Type	Samples	Positive	Mean (µg/kg)	Range (µg/kg)	Country	Author
Black pepper			AFB1 (69.28	84.09	Qatar	Hammami et al. [34]
Chili powder		66.6%	AFB1 (37.44)	5.60 to 69.28	China	Zhao, et al. [35]
Black cumin		40.0%	AFB1 (25)	20 to 30		
Chili		100%	AFs (33.8)	1.9 to 65.7	Spain	Santos et al. [36]
Chili			AFB1 (41)	1.6 to 80.4	Turkey	Bircan [37]
Red pepper	75	72 (96%)	AFB1 (12.405)	0.11 to 24.7	Turkey	Ardic et al. [38]
Pepper	11	5 (54.5%)	AFB1 (13.74)	0.57 to 26.9	Italy	Romagnoli et al. [39]
Pepper	30	13	18.7	1.9 to 35.5	Turkey	Colak et al. [40]
Ground chili		39.7%	18.5		Pakistan	Iqbal et al. [27],
Whole chili		33.3%	17.4			
Ginger (Dry season)		46%	AFs (1.18)	0.17 to 12.02	Nigeria	Lippolis et al. [41]
Ginger (rainy season)		81%	AFs (3.13)	0.11 to 9.52		
Cayenne		25%	0.06		Ireland	O’Riordan and Wilkinson [42]
Coriander		11.1%	0.31			
Paprika		20%	0.32			
Turmeric		40%	1.90			
Ginger	7	1 (14.2%)	0.295		China	Zhao et al. [35]
Mixed Spices	6	5 (83.3)	2.64			
Spices	130	20	0.96	0.59 to 5.38	Italy	Prelle et al. [43]
Chili		37.5%	3	1 to 5	Portugal	Martins et al. [44]

**Table 4:** Estimation of dietary intake for AFs in spices in local population from Punjab, Pakistan.

Product Type	Consumption kg/day	Winter				Summer			
		Afs’ Mean Level µg/kg	Dietary Intake ng/kg/day	MOE	Cancer Risk (Per 10^5^ Individuals Per Year)	Afs’ Mean Level µg/kg	Dietary Intake (ng/kg/day)	MOE	Cancer Risk (Per 10^5^ Individuals Per Year)
Black pepper	0.005	11.2	0.80	0.002	8.4 × 10^−3^	8.8	0.63	0.002	6.6 × 10^−3^
Red pepper	0.01	17.9	2.56	0.006	2.68 × 10^−2^	12.5	1.79	0.004	1.88 × 10^−^^2^
Cloves	0.005	8.4	0.60	0.002	6.3 × 10^−3^	6.5	0.46	0.001	4.87 × 10^−3^
Ginger	0.015	17.6	3.77	0.009	3.96 × 10^−3^	11.45	2.45	0.006	2.58 × 10^−2^
Fenugreek Seeds	0.005	10.2	0.73	0.002	7.65 × 10^−3^	8.6	0.61	0.002	6. 45 × 10^−3^
Garlic powder	0.01	11.1	1.59	0.004	1.76 × 10^−^^2^	9.2	1.31	0.003	1.38 × 10^−2^
Garlic	0.015	11.2	2.40	0.006	2.52 × 10^−2^	8.5	1.82	0.005	1.91 × 10^−^^2^
Onion powder	0.02	11.9	3.40	0.009	3.57 × 10^−2^	10.2	2.91	0.007	3.06 × 10^−2^
Sesame	0.01	12.4	1.77	0.004	1.86 × 10^−2^	9.1	1.30	0.003	1.37 × 10^−2^
Cumin Seeds	0.015	11.9	2.55	0.006	2.68 × 10^−2^	10.5	2.25	0.006	2.36 × 10^−2^

The average weight of male = 70 kg (mean age, 30.5 years).

## Data Availability

The data will be available for request.
